# The Specificity of Downstream Signaling for A_1_ and A_2A_R Does Not Depend on the C-Terminus, Despite the Importance of This Domain in Downstream Signaling Strength

**DOI:** 10.3390/biomedicines8120603

**Published:** 2020-12-13

**Authors:** Abhinav R. Jain, Claire McGraw, Anne S. Robinson

**Affiliations:** 1 Department of Chemical and Biomolecular Engineering, Tulane University, New Orleans, LA 70118, USA; abhinavrjain@gmail.com (A.R.J.); clairemcgraw90@gmail.com (C.M.); 2 Department of Chemical Engineering, Carnegie Mellon University, Pittsburgh, PA 15213, USA

**Keywords:** yeast pheromone response, G protein-coupled receptors, adenosine receptor, C-terminus, G protein, receptor chimera

## Abstract

Recent efforts to determine the high-resolution crystal structures for the adenosine receptors (A_1_R and A_2A_R) have utilized modifications to the native receptors in order to facilitate receptor crystallization and structure determination. One common modification is a truncation of the unstructured C-terminus, which has been utilized for all the adenosine receptor crystal structures obtained to date. Ligand binding for this truncated receptor has been shown to be similar to full-length receptor for A_2A_R. However, the C-terminus has been identified as a location for protein-protein interactions that may be critical for the physiological function of these important drug targets. We show that variants with A_2A_R C-terminal truncations lacked cAMP-linked signaling compared to the full-length receptor constructs transfected into mammalian cells (HEK-293). In addition, we show that in a humanized yeast system, the absence of the full-length C-terminus affected downstream signaling using a yeast MAPK response-based fluorescence assay, though full-length receptors showed native-like G-protein coupling. To further study the G protein coupling, we used this humanized yeast platform to explore coupling to human-yeast G-protein chimeras in a cellular context. Although the C-terminus was essential for Gα protein-associated signaling, chimeras of A_1_R with a C-terminus of A_2A_R coupled to the A_1_R-specific Gα (i.e., Gαi1 versus Gαs). This surprising result suggests that the C-terminus is important in the signaling strength, but not specificity, of the Gα protein interaction. This result has further implications in drug discovery, both in enabling the experimental use of chimeras for ligand design, and in the cautious interpretation of structure-based drug design using truncated receptors.

## 1. Introduction

G-protein coupled receptors (GPCRs) are the largest family of membrane proteins, with over 800 genes in humans [1]. GPCRs are characterized by seven alpha-helical transmembrane domains and bind to extracellular molecules, activating downstream signaling responses inside the cell. GPCRs are found in eukaryotic systems from yeast to mammals and aid in essential functions; in yeast, they mediate the mating pheromone response pathway [2]. Because of their membrane localization and the ability to produce intracellular changes, they are desirable targets for therapeutics, with approximately 40% of drugs on the market targeting these receptors [3,4].

Biophysical characterization and high-resolution structure determination are routinely used for GPCR drug design and discovery and require heterologous expression and purification [5,6,7]. Most receptors are not expressed in heterologous systems at mg/L concentrations required for this structural characterization [8]. Therefore, additional strategies like truncations to remove unstructured regions, thermostabilization via point mutations, and chimeras with thermostable proteins have been utilized to improve expression and crystallization (for example, see modifications for adenosine receptors in Table 1). However, these modifications may change receptor activity and function.

Adenosine receptors are a GPCR subfamily of four receptors (A_1_R, A_2A_R, A_2B_R and A_3_R) that recognize the natural ligand adenosine, an important energy metabolite [39,40]. Adenosine is produced in tissues under stressful conditions like ischemia or hypoxia or energy “demand-supply” imbalance [41,42]. All four adenosine receptor subtypes provide critical protection under stressful conditions and, therefore, are therapeutic targets for Parkinson’s disease, Alzheimer’s disease, cardiovascular diseases, and many others [43]. Multiple crystal structures of A_2A_R have been resolved with bound agonists or antagonists (Table 1). All structures reported for the adenosine receptors contain a C-terminal truncation, except a recently published cryo-EM structure of A_1_R [38].

The C-terminus of A_1_R is 34 amino acids long, whereas the A_2A_R C-terminus is relatively long with 122 amino acids. The two crystal structures of A_1_R contain a truncation from residues 311 and 316. Most crystal structures of A_2A_R contain a truncation from residue 316 (A_2A_Δ316R), corresponding to only 26 amino acids out of the 120, or approximately 20% of the total A_2A_R C-terminus. The long C-terminus of A_2A_R has been hypothesized to be involved in receptor expression [44,45,46], interactions with other signaling partners [47,48], oligomerization [49] and receptor turnover [50,51]. However, previous studies have suggested that the A_2A_Δ316R has native-like signaling [52,53,54] and native-like ligand binding [52,55]. In addition, the absence of the canonical cysteine for palmitoylation (position 309) in A_2A_R has been noted to potentially add flexibility to bind interaction partners [48]; however, the truncation does not appear to alter its desensitization or turnover, as the critical Thr298 is still present [52,56].

Receptor chimeras have been used traditionally to understand the role of the receptor domains in improving functional expression, ligand recognition, and G-protein coupling and specificity, and the ability to produce downstream signaling [46,57,58,59,60]. In our previous study [44], we created an adenosine A_1_/A_2A_ receptor chimera to improve membrane localization and expression in yeast for A_1_ receptor (A_1_R) variants and reported exceptional yields of the active receptor compared to parental A_1_R expressed in any host system to date. In that study, plasma membrane trafficking of A_2A_R and A_2A_Δ316R were similarly efficient, while trafficking of the A_1_/A_2A_R chimera was improved relative to wild-type A_1_R, with resulting improvements in radioligand binding activity, as measured in whole cells [44].

Yeast shares many functionally exchangeable proteins involved in the GPCR signaling pathway with higher eukaryotes [2,61], has served as a useful microbial platform for rapid ligand screening and leads the development of orphan GPCRs [62]. The most researched GPCR-mediated pathway in yeast is responsive to the presence of peptide mating pheromones that regulate metabolism related to mating. Activated receptors catalyze dissociation of Gpa1, the yeast G protein, activating a mitogen-activated protein kinase (MAPK) cascade, which has been used as a unique platform to study human GPCR signaling [63]. In contrast, the presence of multiple GPCRs and Gα proteins in native mammalian systems can confound the results from downstream signaling assays. Yeast provides a relatively simple and inexpensive platform without the complexities of multiple GPCRs, receptor promiscuity, and crosstalk that occurs in native mammalian hosts [64,65].

**Figure 1:**
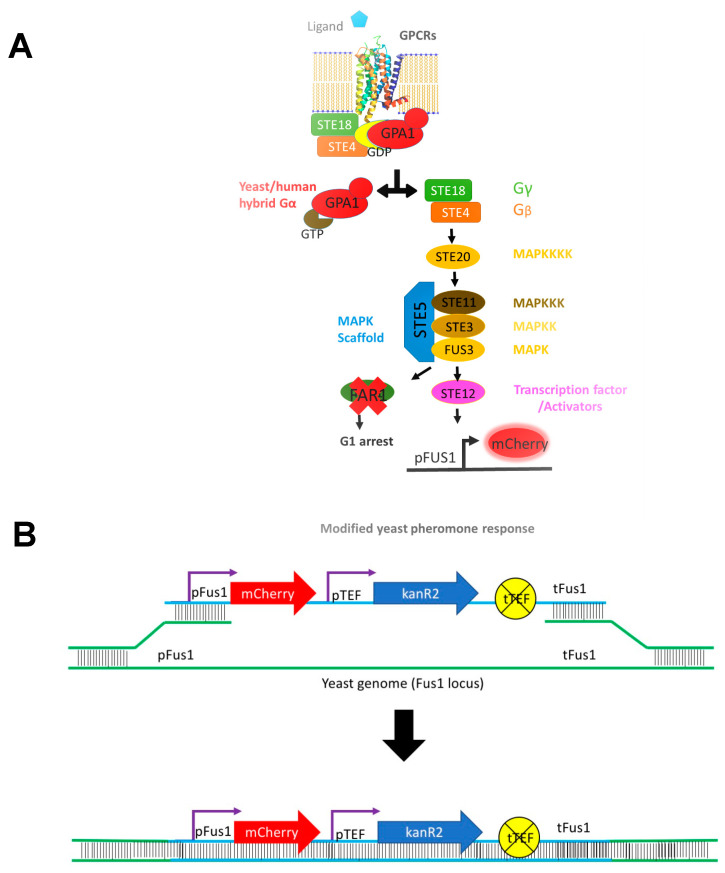
(**A**) Schematic of G-protein coupled receptors (GPCR)-mediated mitogen-activated protein kinase (MAPK) signaling cascade in yeast. In this engineered pheromone response signaling pathway, cells express a yeast/human chimeric Gα protein to enable human GPCRs to couple with the yeast signaling pathway. Upon activation of downstream signaling, cells express mCherry fluorescent protein, which acts as an indirect measure of receptor activation. (**B**) Diagram represents homologous recombination approach used to include the mCherry gene along with the antibiotic resistance gene for clone selection within the *Fus1* locus of yeast strains.

Engineered yeast strains with modification to the native MAPK-based signaling pathway to report on ligand-mediated downstream signaling from human GPCRs (Figure 1A) were obtained both from the Broach laboratory [66] and the Dowell laboratory at GlaxoSmithKline [67]. In these yeast strains, the last five amino acids of native yeast Gα (Gpa1) were replaced with the last five amino acids residues from a human Gα to yield native-like GPCR-Gα interactions. This replacement has been shown to be sufficient for coupling with many human GPCRs, including human A_2A_R, resulting in a native-like dose response and ligand binding order preference [67]. Because of structure-based drug discovery efforts that rely on truncated receptors for in silico screening, we investigated one of the key protein-protein interactions of the C-terminus, coupling to G-protein to activate downstream signaling by utilizing this engineered yeast pheromone response pathway.

## 2. Experimental Section

### 2.1. Materials

Adenosine receptor ligands 5′-N-ethylcarboxamidoadenosine (NECA), N^6^-cyclopentyladenosine (CPA) and CGS21680 were purchased from Tocris (Minneapolis, MN, USA). Forskolin was obtained from Sigma-Aldrich (St. Louis, MO, USA). Precision Plus Protein Western C Standards was purchased from Biorad (Hercules, CA, USA). Human embryonic kidney cells (HEK-293, were obtained from ATCC (Manassas, VA, USA). Dulbecco’s modified eagle medium (DMEM, 11995-065), Opti-MEM I reduced serum media (31985-070), fetal bovine serum (FBS, 16000-044), Lipofectamine 2000 transfection reagent (11668-019), RIPA buffer, Halt Protease and Phosphatase Inhibitor Cocktail, mammalian expression vectors (pCEP4) and Alexa 568-donkey anti-rabbit antibody (A10042) were obtained from Invitrogen Life Technologies (Carlsbad, CA, USA). The cAMP dynamic 2 kit was purchased from Cisbio US Inc. (Bedford, MA, USA). The mouse monoclonal A_2A_R antibody was obtained from Santa Cruz Biotechnology (sc-32261, Dallas, TX, USA). The rabbit anti-GFP antibody (ab6556) and goat pAb to Mouse IgG HRP antibody (ab97265) were obtained from Abcam (Cambridge, MA, USA).

### 2.2. Strains and Culture Conditions

*E. coli* strain DH5α was used for amplifying yeast expression plasmids and mammalian expression vectors. *E. coli* was grown in Luria-Bertani media supplemented with 100 μg/mL ampicillin at 37 °C at 250 rpm.

All yeast strains used in this study are summarized in Table 2. Yeast strains with modified pheromone response pathway and human-yeast chimeric Gα proteins (Figure 1A) were obtained from the Broach laboratory [66] and Glaxo-Smith-Kline (GSK) [67]. These parental yeast strains were grown in YPD media (2% bacto peptone, 2% glucose, 1% yeast extract) and, depending on the fus1 transformation, supplemented with 300 µg/mL hygromycin B or 200 µg/mL G418. Yeast expression plasmids were constructed using homologous recombination in *S. cerevisiae* strain BY4741 (*MATa his3*Δ*1 leu2*Δ*0 met15*Δ*0 ura3*Δ*0*) and were grown in synthetic media. The synthetic media (SD or SG) was comprised of 2% dextrose or galactose, respectively, 0.67% yeast nitrogen base, citrate buffer at pH 5.4 (4.2 g/L citric acid and 14.7 g/L sodium citrate) and supplemented with amino acids and essential nutrients as described by Burke and colleagues [68]. Uracil was omitted from this media (SD-ura or SG-ura) to select for plasmid-containing cells. Yeast was grown in culture tubes and multiwell plates at 30 °C at 275 rpm.

Human embryonic kidney (HEK-293) cells were maintained in growth media containing DMEM with 10% FBS at 37 °C in a 5% CO_2_ incubator. Transient transfections were performed by seeding cells on day 0 to be approximately 70% confluent on day 1. On day 1, cells were transfected using 10 μL Lipofectamine 2000 reagent, and 1 μg DNA in 2 mL Opti-MEM reduced serum media (per 25 cm^2^ flask). On day 2, the media was replaced by FBS supplemented DMEM media, and used for experimentation on day 3, approximately 36 h post-transfection. The cAMP accumulation assay described below (Section 2.6) was performed on cells with a passage number lower than 25.

### 2.3. Yeast Genomic Transformation

To develop a fluorescence-based assay to measure the downstream signaling response in yeast following ligand binding, monomeric Cherry fluorescent protein (mCherry) [69] was introduced into the *FUS1* locus under control of the FUS1 promoter. To this end, overlapping fragments were first assembled in yeast using a homologous recombination as described below. The fragment consisted of the mCherry fluorescent protein and hygromycin resistance gene *hphMx6* or kanamycin resistance gene *KanR2* with the translation elongation factor 1 promoter and terminator (pTEF and TEFt). The fragment was flanked with approximately 300 base pairs of the Fus1 promoter and Fus1 terminator to aid in genomic recombination. The Fus1 promoter and Fus1 terminator sequences were amplified from BY4741 using colony PCR. The mCherry protein and pTEF-hphMx6-TEFt fragments were amplified from the pBS35 plasmid, while the pTEF-KanR2-TEFt fragment was amplified from the pBS7 plasmid (Figure 1B). Both pBS7 and pBS35 were obtained from the Yeast Resource Center at the University of Washington. The fragments were assembled in BY4741 using a homologous recombination using pRS316 as a template. Fragment assembly was verified using Sanger sequencing (Operon, Louisville, KY, USA). The resulting fragment was then amplified using PCR and transformed into yeast using the protocol from Gietz and Woods [70]. Colony PCR was used to confirm successful genomic integration. Partial sequencing confirmation of final clones was obtained for some of the transformants.

### 2.4. Subcloning and Plasmid Construction

A set of yeast expression plasmids (Table 3) containing a GPCR and C-terminal protein tags, necessary for adenosine receptor detection and quantification, was constructed using a homologous recombination in BY4741 as described previously [44]. The plasmid contains a galactose (pGAL_1-10_) promoter, a pre-pro leader sequence (PP) [71] for targeting to the secretory pathway and the CYC1 terminator (CYC1_t_) [44]. For fluorescence microscopy, the GPCRs were C-terminally tagged for easier detection of protein expression with monomeric Citrine fluorescent protein (mCitrine) [44,57,58,72]. Sequencing was used to confirm the correct gene sequence for the constructs (Operon, Louisville, KY, USA).

Mammalian expression vector pCEP4 was used for expressing receptors in transiently transfected HEK-293 cells. Untagged A_1_R and A_1_/A_2A_R were inserted into the pCEP4 multiple cloning site between HindIII and NotI restriction enzyme sites, whereas A_1_/A_2A_∆316R, A_2A_R, and A_2A_∆316R were inserted between KpnI and XhoI restriction enzyme sites. Transformations of *E. coli* were performed by the heat shock method. Sequencing was used to confirm the correct gene sequences for the plasmids (Operon, Louisville, KY, USA).

### 2.5. Cyclic Adenosine Monophosphate Accumulation Assay

Transiently transfected HEK-293 and either non-transfected cells or cells transfected with empty plasmid (control) were incubated for 30 min in the presence or absence of ligand (DMSO only, no ligand control) at a cell density of 1000 cells/well in a white 384-well plate (Grenier Bio-One #784075, Monroe, NC, USA). Excess cells were pelleted and stored at −80 °C for subsequent Western blotting. The concentration of cAMP per well was determined using the cAMP dynamic 2 kit using a BioTek Synergy H1 Plate Reader according to the manufacturer’s protocol. Our previous study [73] has shown that adenosine deaminase (ADA) pre-treatment of cells did not alter the ligand binding or downstream signaling, and therefore the cells were not treated with ADA prior to ligand treatment while utilizing the CisBio HTRF kits. Experiments were performed in triplicate for three independent transfections. Data were analyzed as per manufacturer’s recommendation, and mean and standard error were plotted using Prism (GraphPad, La Jolla, CA, USA). Student’s *t*-test was performed using Prism to obtain the significance of the data.

### 2.6. MAPK Response Signal Determination

Yeast cultures were grown overnight in SD-ura selection in 400 µL or 1 mL media in 48-well or 24-well plates (Falcon 353047 and 353078, Corning, NY, USA), respectively, at 30 °C at 275 rpm. Recombinant GPCR expression was induced by transferring 12.5 µL of overnight culture into 400 µL SG-ura. For some strains, 0.125% glucose was used to supplement the SG-ura media to improve cell growth of the engineered yeast strains. This level of glucose supplementation has been shown to result in minimal glucose-based suppression of the galactose promoter, as described previously [74]. Yeast cells expressing the receptor were imaged using Nikon A_1_ laser-scanning confocal microscope, as described in our earlier published study [44].

To determine signaling, all ligand stock solutions were prepared to the highest soluble concentration (typically around 40–100 mM) in dimethyl sulfoxide (DMSO), according to the recommendations of the manufacturer. Working concentrations of 5 mM ligand (50X) in DMSO were used for all yeast signaling experiments. After 24 h of GPCR expression, twelve µL of the overnight culture was added to 380 µL fresh SG-ura media per well of a 48-well plate. Eight microliters of ligand or DMSO were added to each well (final DMSO concentration at 2% (*v*/*v*) per well). Although this concentration is well above the K_D_, a high ligand concentration has been shown previously to be needed for effective downstream signaling response in yeast [75,76,77,78]. After ligand addition, the 48-well plate was incubated at 30 °C at 275 rpm for 24 h. Adenosine deaminase treatment was not required for working with the yeast-based assay, as previous studies show this treatment does not impact downstream signaling measurements [79,80]. Similar results were obtained from 4 h incubations, but the fold change difference was not as pronounced. Fluorescence intensities of 100 µL of resulting liquid culture were measured in triplicate in a 96-well plate (Costar 3915, Corning, NY, USA) using a BioTek Synergy H1 microplate reader (Winooski, VT, USA) maintained at 30 °C. Experiments were performed for six independent transformants.

### 2.7. Western Blotting

Transiently transfected HEK-293 cells were scraped, pelleted, and resuspended in ice-cold 1X TE buffer (1% 1M Tris-Cl pH 7.5, 0.2% 500 mM EDTA pH 8) with protease inhibitors. Cells were sonicated with a Branson Sonifier 450 at 50% power for 30 pulses and then centrifuged at 2000× *g* for 5 min at 4 °C to remove cell debris and unlysed cells. The supernatant was then centrifuged at 100,000× *g* for 1 h at 4 °C to pellet cell membranes. Membranes were solubilized in 1X RIPA buffer (10 mM Tris-Cl (pH 8.0), 1 mM EDTA, 0.5 mM EGTA, 1% Triton X-100, 0.1% sodium deoxycholate, 0.1% SDS, 140 mM NaCl) with protease inhibitors; if necessary, membranes were sonicated again for five pulses at 50% power to break up any visible pieces of membrane. BCA assay (Pierce; Rockford, IL, USA) was performed to determine the total protein concentration of isolated membrane, using bovine serum albumin (BSA; Thermo Fisher, Waltham, MA, USA) as a standard.

Isolated HEK cell membranes were utilized for A_2A_R and A_2A_Δ316R protein quantification via Western immunoassay. Western blotting analysis could not be performed for untagged A_1_R and its variant due to the lack of an effective antibody against the receptor. The untagged (non-fluorescent) receptor was used for the study to not interfere with the fluorescence-based CisBio HTRF kits. A total of 10 μg of total protein per sample was loaded onto a 12% Tris-Glycine gel and electrophoresed in SDS buffer at 125 V for 65 min. Western immunoassay was performed using adenosine A_2A_R mouse monoclonal IgG antibody (sc-32261, Santa Cruz Biotechnology, Dallas, TX, USA) at 1:5000 dilution, and Goat pAb to Mouse IgG HRP antibody at 1:5000 dilution. Membranes were imaged with the UVP BioSpectrum imaging system.

Yeast cell pellets (10 OD_600_) were resuspended in 250 µL lysis buffer (10% glycerol, 50 mM sodium phosphate, 300 mM sodium chloride, pH 8) supplemented with cOmplete EDTA-free protease inhibitor cocktail (Roche, Indianapolis, IN, USA). An equal volume of 0.5 mm zirconia/silica beads (BioSpec, Bartlesville, OK, USA) was added to the cells and lysis was performed using a vortexer or a BeadBug homogenizer (Benchmark Scientific, Edison, NJ, USA). Cell lysates were combined with 4X Laemmli sample loading buffer supplemented with β-mercaptoethanol (Bio-Rad, Hercules, CA, USA). One OD_600_ equivalent of cell lysate was loaded per well for Western blotting. Precision Plus Protein WesternC Standard (BioRad) was used as a standard to enable molecular weight estimation. Rabbit anti-GFP antibody (1:5000 dilution) and Alexa 568-donkey anti-rabbit (1:2500) was used to detect mCitrine protein-tagged receptors.

## 3. Results

### 3.1. Loss of the Cytoplasmic C-Terminus Results in Loss of Downstream Signaling

The A_2A_R truncation at residue 316 (A_2A_Δ316R) present in many crystal structures (Table 1) contains helix 8 and some residues of the cytoplasmic tail, and has been reported previously to have native-like affinity for the agonist NECA and antagonist ZM 241385 in mammalian and yeast systems [52,55,81]. In prior studies, we showed that the full-length and truncated A_2A_R (A_2A_Δ316R) receptors trafficked to the yeast plasma membrane comparably and showed similar ligand-binding ability [44].

**Figure 2:**
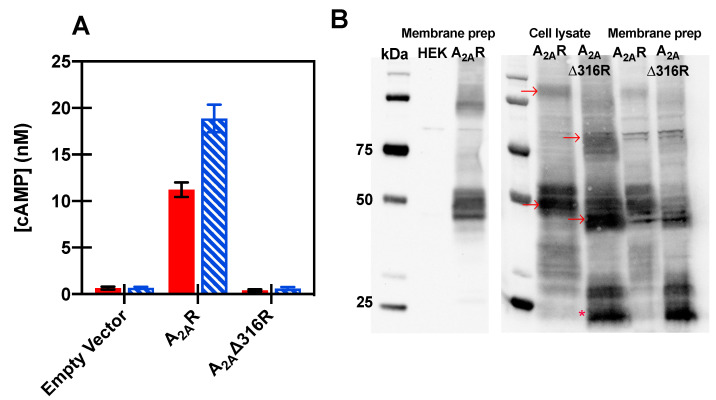
Transiently transfected HEK-293 cells were used to determine downstream signaling for chimeras. (**A**) Agonist-mediated cAMP accumulation for transiently transfected cells with A_2A_R and A_2A_Δ316R (DMSO only shown in red filled bars, 1 μM CGS21680 agonist in blue hatched bars). Data represent mean ± S.E.M. for three independent transfections performed in triplicate (* *p* < 0.001, Student’s *t*-test). (**B**) Representative Western blot analysis of A_2A_R and its truncation from transiently transfected in HEK-293, as obtained from equal protein loadings of total cell lysate or membrane fractions. HEK sample shows HEK-293 cells transfected with pCEP only compared to pCEP-A_2A_R. Dimer and full-length monomeric receptor are indicated by arrows in cell lysates, and multiple forms visible—particular in the monomeric form at ~50 kDa—can be attributed to variable glycosylation. Samples of A_2A_Δ316R shows a smaller band visible at ~30 kDa that is likely a proteolytic product, indicated by an asterisk. Expected molecular weights for A_2A_R is 44.7 kDa and A_2A_Δ316R is 35.1 kDa, and molecular weights were determined using Precision Plus Protein Western C standards.

Here, HEK-293 cells were transiently transfected with pCEP4 encoding full-length or truncated receptor in order to determine the effect of the truncation on downstream signaling. A_2A_R couples to Gsα, and thus agonist addition activates adenylyl cyclase, resulting in increased cAMP synthesis. As expected, cells transfected with empty plasmid showed negligible cAMP synthesis in the absence of ligand and remained unchanged following the addition of a selective A_2A_R agonist, CGS21680 (1 μM) (Figure 2A). The presence of full-length A_2A_R led to constitutive activation in the absence of ligand as well as a significant increase in cAMP levels following agonist treatment, consistent with previous studies [73]. Surprisingly, A_2A_Δ316R showed no increase in cAMP levels upon agonist addition (Figure 2A). The loss of signaling for the truncated A_2A_Δ316R was surprising, as the agonist-bound crystal structures have been reported to be in an active state [11,18], and the truncation localizes well to the cell surface (reported by our lab previously in [44]). This A_2A_R truncation at residue 316 has been reported previously to have native-like binding to the agonist NECA and the antagonist ZM 241,385 by Magnani and colleagues [81] at 32 and 12 nM, respectively, compared to 20 and 1 nM for the full-length receptor [82]. Furthermore, we find that A_2A_Δ316R also showed no constitutive activity, though this construct contains residues Y197 and Y288 and NPxxY in TM7 that have been reported to be important for binding G protein in active structures [83]. These data show that though A_2A_Δ316R binds ligand, it does not activate Gsα, suggesting the C-terminus is necessary for downstream signaling of the receptor.

Western blot analysis of membrane preparations was utilized to verify that the absence of A_2A_Δ316R activity was not due to a lack of protein expression. Though interpretation of Western quantitation should be undertaken with caution, it is clear that there are no significant differences in the A_2A_R and A_2A_Δ316R expression in HEK-293 cells (Figure 2B), suggesting the >20-fold differences seen in cAMP signaling were not the result of expression differences. Cells transfected with pCEP control vector did not show any receptor expression via Western blot analysis (Figure 2B) and previous work from our laboratory has shown that the multiple bands (particularly evident in the monomeric form) are the result of protein glycosylation that occurs in the HEK cells [84].

**Figure 3:**
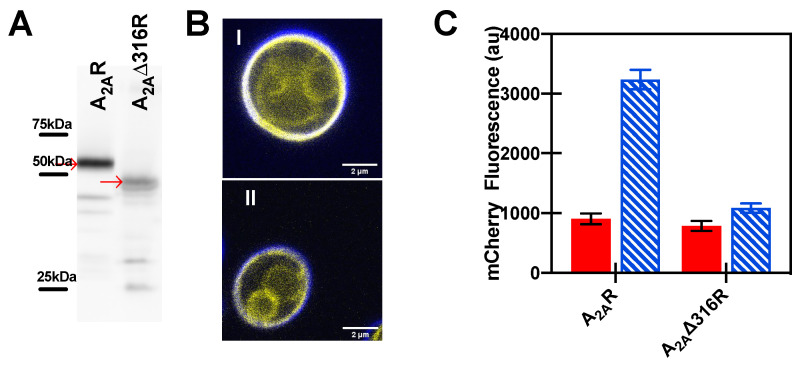
Truncation of the C-terminus for A_2A_R (A_2A_Δ316R) resulted in a loss of the signaling response in yeast strains. (**A**) Full-length expression of mCitrine tagged wild-type and truncated A_2A_R was observed using Western blot analysis with an anti-GFP antibody. Full-length receptor is indicated by an arrow. Molecular weights were estimated using Precision Plus Protein Western C standards. The expected molecular weight of each receptor is as follows: A_2A_R, 71.6 kDa; and A_2A_Δ316R, 66.1 kDa. Note that the mobilities are slightly faster than that expected by calculated molecular weight; membrane proteins have been found to run faster than expected, possibly due to their hydrophobicity or incomplete denaturation, due to the absence of sample heating [88]. (**B**) Representative confocal images of yeast cells expressing (i) A_2A_R and (ii) A_2A_Δ316R show plasma membrane localization of the receptor. Scale bar = 2 µm. Images performed as described in Experimental Section. (**C**) MAP kinase response signaling of the full length A_2A_R and truncated (A_2A_Δ316R) receptor in Gpa1p-Gαs engineered strain. NECA (100 μM) shown as blue hatched bars and DMSO control as red filled bars. Data represents the mean ± 95% C.I. for experiments performed in duplicate for three independent transformants.

We further examined the expression of full-length A_2A_R and A_2A_Δ316R with C-terminal tagged mCitrine fluorescent protein fusions in yeast. Expression was confirmed using Western blot analysis (Figure 3A). Previous studies from our laboratory have shown that the C-terminal fluorescent protein fusion does not impact trafficking or downstream activation of A_2A_R [55,85,86,87], and we previously reported that full-length and truncated A_2A_R (A_2A_Δ316R) receptor trafficked to the yeast plasma membrane comparably and showed similar ligand binding ability [44]. Confocal microscopy shows the localization of both full-length and truncated A_2A_R primarily at the plasma membrane (Figure 3B).

As described in the Experimental Section, engineered yeast strains with modification to the native MAPK-based signaling pathway [66,67] were further modified to replace the original Fus1 modification that relied on growth-dependent signaling (via His3 expression) in the Broach lab strain [66] or β-galactosidase reporter activity in the GSK strains [67] with an easily detectable fluorescence signal from monomeric Cherry (mCherry) fluorescent protein (Figure 1 and Table 2). Because the yeast G protein-coupled signaling pathway contains homologues to proteins in the human signaling pathway, engineered yeast have been used to successfully recapitulate native ligand-binding preferences and G protein-coupling for human GPCRs [66,67,79,80]. In these engineered yeast strains, mCherry is produced in the cells upon ligand-mediated downstream signal activation via human GPCR-Gα protein coupling (Figure 1A). The fold change in mCherry fluorescence in these strains can be easily compared by the addition of agonist relative to a control. As in HEK cells, upon agonist addition, there was no downstream signaling observed in the truncated A_2A_Δ316R as compared to the full-length receptor in the stimulatory yeast strain (Figure 3C). This observation is important as it allows us to use the yeast system to screen and validate receptor variant activity.

### 3.2. The C-Terminus Does Not Play a Role in the Specificity of Gα Coupling

In order to understand how the C-terminus of A_2A_R might be involved in the specificity of Gα coupling, we utilized an adenosine A_1_/A_2A_ receptor chimera, where the construct contains all seven transmembrane domains of A_1_R (residues 1–290) and the C-terminus of the A_2A_R (residues 291–412) created using homologous recombination [44]. In addition, a truncated chimera (A_1_/A_2A_Δ316R) consisting of the transmembrane domains of A_1_R and the C-terminus of A_2A_R truncated at the 316th residue was constructed, consistent with the truncated A_2A_Δ316R utilized in crystallography. This truncated chimera contains the helix 8 residues of A_2A_R. In our previous study, trafficking of the A_1_R/A_2A_R chimera was improved relative to wild-type A_1_R, with resulting improvements in radioligand binding activity to the A_1_-selective ligand [^3^H]-DPCPX, as measured in whole cells [44]. The K_D_ for DPCPX determined for A_1_/A_2A_R and A_1_/A_2A_Δ316R were 4.8 and 7.3 nM, respectively [44], which are consistent with the reported values for A_1_R with DPCPX (0.18–6.1 nM) [89,90,91,92].

**Figure 4:**
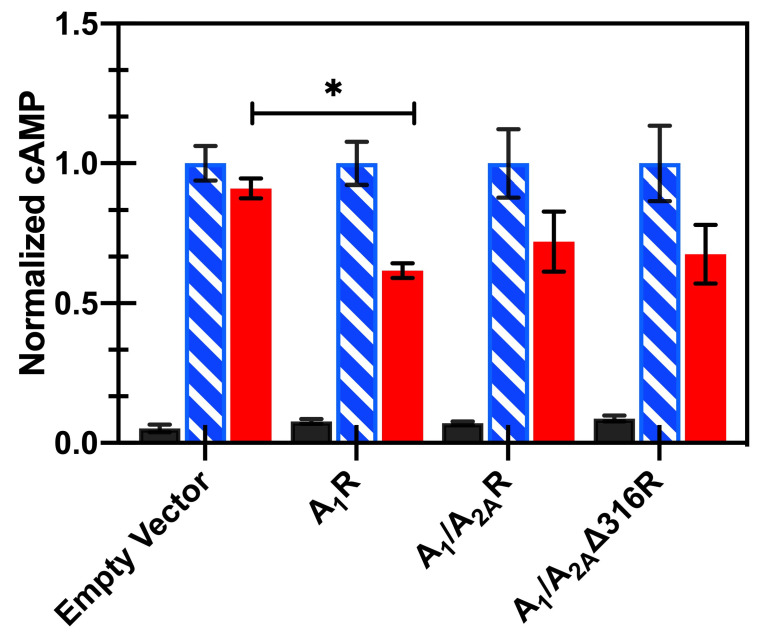
Expression of A_1_R or A_1_/A_2A_R chimeras in HEK-293 cells results in inhibition of cAMP production after forskolin stimulation. Inhibition of cAMP production with A_1_R and compared to pCEP (empty) vector controls and A_1_/A_2A_R and A_1_/A_2A_Δ316R chimeras (no ligand in black filled bars, 10 μM forskolin in blue hatched bars, 10 μM forskolin and 1 μM CPA in red filled bars). Data represent the mean ± S.E.M. for three independent transfections performed in triplicate (* *p* < 0.001, Student’s *t*-test).

To test the role of the A_2A_R C-terminus in A_1_R signaling in mammalian cells, cAMP was measured in transiently transfected HEK-293 cells. A_1_R couples to Gαi/o, which inhibits activation of adenylyl cyclase, so in the absence of ligand there should be minimal changes to cAMP levels, consistent with our results (Figure 4). Forskolin directly activates adenylyl cyclase, which leads to stimulation of the production of cAMP even in cells not expressing A_1_R, so treatment with 10 μM forskolin was used to elevate the basal level of cAMP. Cells expressing A_1_R showed a reduction in cAMP following treatment with an A_1_R-selective agonist (1 μM CPA in the presence of 10 μM forskolin).

Next, we compared cAMP activation in HEK cells for wild-type A_1_R with the A_1_/A_2A_R and A_1_/A_2A_Δ316R chimeras. The addition of the A_2A_R C-terminus to A_1_R did not lead to constitutive activity of the receptor in the absence of a ligand (Figure 4, black filled bars); therefore, 10 μM forskolin was used to enable a basal cAMP signal. When treated with the A_1_R-specific agonist CPA (1 µM, in the presence of 10 μM forskolin), cells transfected with either A_1_/A_2A_R or A_1_/A_2A_Δ316R showed a moderate reduction in cAMP signaling (72 ± 10% and 67 ± 10%, respectively), consistent with the wild-type A_1_R (62 ± 3%), verifying that A_1_/A_2A_R and A_1_/A_2A_Δ316R chimeras couple to Gαi (Figure 4, red filled bars). Note that the data were normalized to cAMP levels for forskolin treated cells for each variant.

**Figure 5:**
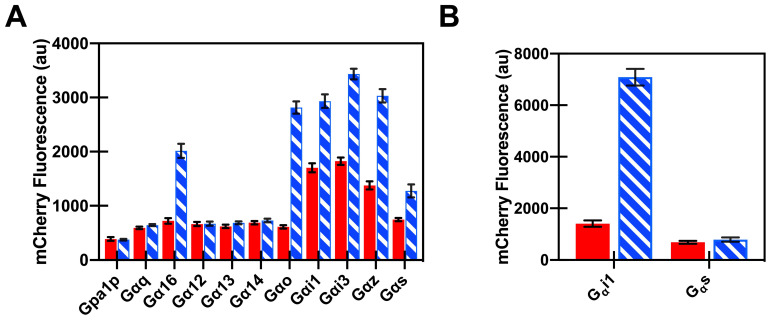
Downstream signaling in engineered yeast cells enables determination of Gα coupling preferences. Agonist (100 μM NECA, blue hatched bars) mediated downstream signaling responses as compared to control (DMSO, red filled bars) for A_1_R were measured in yeast expressing Gpa1p-human Gα chimeras ( mean ± S.D., for three independent experiments) for either modified GSK strains (**A**) or Broach laboratory strains (**B**).

To recreate a cellular library of human GPCR downstream signaling, twelve strains containing different yeast-human Gα chimeras that reproduce downstream signaling responses of human Gα proteins [67] were modified, as described in Section 3.1 above, to enable MAPK-activated increases in mCherry fluorescence. These strains can be classified into five Gα families: Gαi/o, Gαs, Gαq, Gα12 and native Gα. First, to ensure full-length human A_1_R showed mammalian coupling behavior in yeast, we mapped the interaction between the A_1_ adenosine receptors and the appropriate Gα in the engineered Gα chimera strains, using the non-selective high-affinity adenosine receptor family agonist, NECA (100 µM, Figure 5A). A_1_R showed a signaling response upon agonist addition with the inhibitory Gα family (Gαi1, Gαi3, Gαo and Gαz) and the promiscuous Gα16 (Figure 5A), as expected for this receptor based on the mammalian preferences for inhibitory Gαs. The highest fold change between cells treated with NECA as compared to DMSO-treated cells was observed for the yeast strain expressing the Gpa1-Gαo chimera. Note that ligand levels used for these studies were well above expected K_D_ values; however, the use of high ligand concentration is consistent with earlier studies [67,79,80,93], and perhaps reflects the reduced ability of hydrophobic ligand to penetrate the chitosan-rich yeast cell wall and then reach the plasma membrane, resulting in an apparent reduced effective ligand concentration at the membrane.

To ensure that our results were not strain-dependent, we compared the signaling response obtained from Gpa1-Gαi1 and Gpa1-Gαs engineered yeast strains obtained from GSK to those modified from those of the Broach laboratory. Consistent with those from the GSK laboratory strains (Figure 5A), A_1_ receptors maintained their native Gα coupling-specificity (Figure 5B). Although both strains are derived from W303 parental yeast cells, interestingly, the Broach laboratory strains showed a higher fold change in mCherry fluorescence, as well as a reduced constitutive activity. Because of the higher fold change in fluorescence compared to the GSK strains, the Broach strains were utilized for subsequent studies.

**Figure 6:**
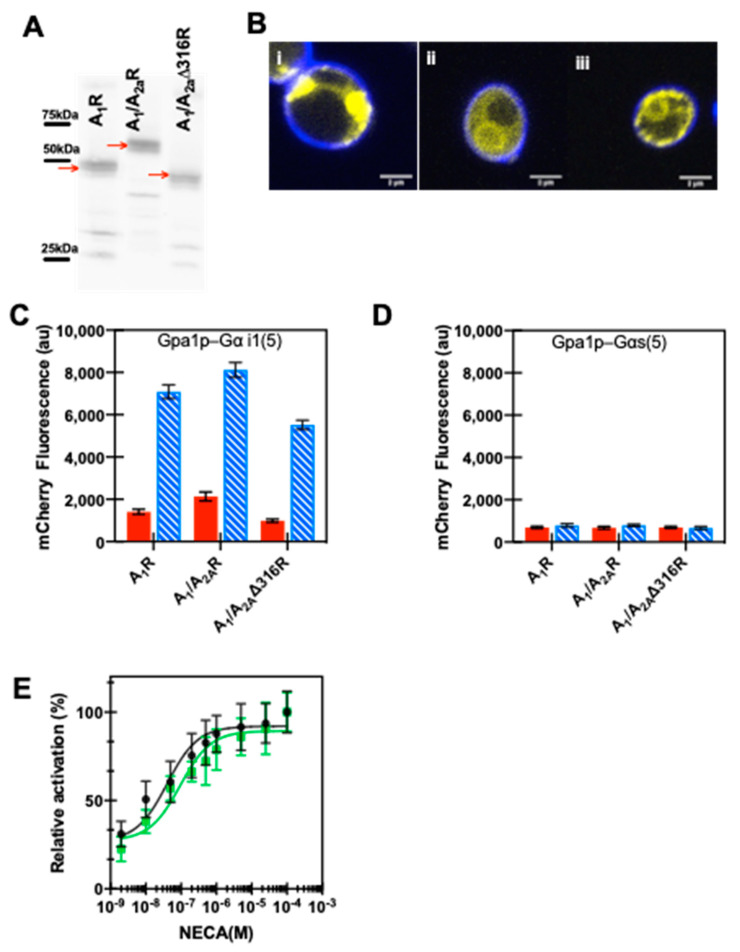
Expression and downstream MAPK signaling response in yeast for A_1_/A_2A_R and A_1_/A_2A_∆316R chimeras show native A_1_R-like behavior. (**A**) Western blot images showing expression of mCitrine tagged receptors for full-length and truncated chimeric receptors. Precision Plus Protein Western C standards were used to determine molecular weight as indicated. The expected molecular weight of each receptor with mCitrine is as follows: A_1_R, 63.4 kDa; A_1_/A_2A_R-71.5 kDa and A_1_/A_2A_Δ316R-62.3 kDa. Note that the mobilities are slightly faster than that expected by calculated molecular weight; membrane proteins have been found to run faster than expected, possibly due to their hydrophobicity or incomplete denaturation, due to the absence of sample heating [88]. (**B**) Representative confocal images of yeast strains showing receptor localization of A_1_R (i), and A_1_/A_2A_R (ii) and A_1_/A_2A_Δ316R (iii). Cells were stained with fluorescent brightener calcofluor white M2R (F3543; Sigma-Aldrich) that binds to chitin in the yeast cell wall prior to imaging, and this stain is shown in blue, while mCitrine fluorescence is shown in yellow. Scale bar = 2 µm. (**C**,**D**) Downstream signaling response of receptor variants in inhibitory Gpa1p-Gα i1(5) (**C**) or stimulatory Gpa1p-Gαs(5) strain (**D**). A_1_/A_2A_R and A_1_/A_2A_Δ316R produce signaling response similar to A_1_R in inhibitory strain; however, none of the receptors produce a response in stimulatory strain. 100 μM NECA is shown as blue hatched bars and DMSO in red filled bars. (**E**) Dose-response curve for A_1_/A_2A_R chimera (green squares) is similar to the native A_1_R receptor (black circles). Data represent the mean ± 95% C.I. for experiments performed in duplicate for three independent transformants.

Expression of the chimeras was observed using Western blot analysis (Figure 6A) and confocal microscopy shows the localization of both full-length and truncated A_1_/A_2A_R chimeras to the plasma membrane (Figure 6Bii and iii) as compared to wild-type A_1_ receptor (Figure 6Bi). The downstream signaling response was evaluated in the inhibitory (Figure 6C) and stimulatory (Figure 6D) yeast reporter strains, and both chimeras showed coupling with the inhibitory yeast strain, similar to wild-type A_1_R, though the A_1_/A_2A_Δ316R chimera showed reduced MAPK signaling via lower mCherry levels than the full-length A_1_/A_2A_R chimera. No signaling response was obtained in the stimulatory yeast strains for the A_1_R variants (Figure 6D). This observation suggests that the presence of the A_2A_R C-terminus does not affect the interaction of the chimeric A_1_/A_2A_ receptors with the native-like inhibitory Gα and that it does not become non-selective, i.e., by binding to all Gα proteins. This observation is consistent with previously published work with canine A_1_R and A_2A_R, where an A_1_R chimera showed native coupling with Gαi/o [60]. We also determined the dose-dependent mCherry fluorescence response for A_1_R and the A_1_/A_2A_R chimera and found that they were statistically equivalent when the non-specific agonist, NECA, was added (Figure 6E).

## 4. Discussion

Since the early 1990s, the engineered yeast MAPK response pathway has been known as a useful tool to study human GPCR signaling and identify lead drug candidates by recapitulating native dose-response binding preferences [63,66,93]. Both A_1_R and A_2A_R have been shown previously to interact with yeast/human chimeric Gα protein to produce downstream signaling responses in the engineered yeast [79,80,93,94]. Here, these yeast strains were further engineered with a fluorescence reporter and also successfully captured A_1_R and A_2A_R downstream signaling via their corresponding native Gα proteins. Strains obtained from the Broach laboratory showed a higher fold change than those from the Dowell (GSK) laboratory under these conditions, although both strains were derived from parental W303 cells, indicating that strain differences outside of the *FUS1* locus can impact the results obtained in cell-based assays.

One of the strengths of the engineered yeast system is the capability of quantifying the GPCR-Gα interaction at a common endpoint of the signaling cascade. This allows direct comparison of the strengths of the activation for different Gα biased ligands. One such study performed by Stewart et al. [93] identified a novel A_1_R agonist with biased specificity for Gαi vs. Gαo coupling. Efforts have been made to replicate this approach of utilizing the last five amino acids of the C-terminus of the Gα protein into a mammalian system using Gαs or Gαq as templates [95,96]. A study by Hsu and Lou [95] in HEK-293 cells tested the interaction of A_1_R with Gαs chimeras via a cAMP assay. The authors observed cAMP production for all Gα variants tested except Gαs, suggesting the system was not effective in capturing the specificity of the interaction of A_1_R with Gα proteins. The higher than native levels of Gα protein expressed (~three-fold higher than mock transfected, native HEK) [97,98] and the presence of additional GPCRs present in the HEK cells may have contributed to signal promiscuity [99]. In our study, all the signaling components in the engineered yeast were expressed under their native promoters and, perhaps as a result, the yeast cell assay more effectively captured the specificity of GPCR-Gα interaction.

The long C-terminus of the A_2A_R (122 amino acids) is assumed to be highly flexible and disordered; thus, crystallization of adenosine receptors has all focused on using truncated receptors. Here, we found that A_2A_Δ316R resulted in no downstream cAMP signaling, and that the A_2A_R C-terminus did not change the G-protein coupling preference from Gαi to Gαs for the A_1_/A_2A_R variants. Our results were consistent with previously published work by Tucker et al. [60] that found that a chimera of canine A_1_R with a canine A_2A_R C-terminus showed no change in G-protein coupling behavior. The A_1_/A_2A_R chimera showed a dose-dependent MAPK response similar to the wild-type human A_1_R receptor in the yeast system, suggesting there was no change in G-protein coupling behavior due to the presence of the A_2A_R C-terminus. Taken together with our previous results of exceptional yields of the chimera that binds A_1_R-selective agonist [44], these data suggest that A_1_/A_2A_R could be an effective variant to study biophysical characteristics and function for the A_1_ receptor. Previously, we have reported that a similar A_3_/A_2A_R chimera, consisting of the A_3_R transmembrane helices and the A_2A_R C-terminus, showed native coupling with Gαi/o but not Gαs [58]. As noted in the introduction, the C-terminus of A_2A_R has some special properties, such as the absence of the canonical cysteine for palmitoylation (position 309 in A_2A_R) that may potentially add flexibility to bind interaction partners [48]. Thus, although these results are of interest for the adenosine receptors family, more experiments will need to be performed to understand the general applicability of this chimeric approach to another class A GPCR subfamily.

The A_2A_R C-terminus is known to interact with many accessory proteins in the GPCR signaling pathways like G protein receptor kinases and β-arrestins that aid in receptor signaling and desensitization [47,48,100], but has previously been thought to be dispensable for G-protein signaling [52,54]. Bennett et al. [53] showed that A_2A_Δ316R expressed by an inducible promoter was capable of coupling to Gαs in a receptor expression-level dependent manner; however, their data were normalized, and total cAMP levels were not reported. We do see a small increase in ligand-dependent signaling for the A_2A_Δ316R truncation (Figure 2A), but the signal is over twenty-fold less than wild type A_2A_R, suggesting the truncation is responsible for the loss of G protein signaling. Taken together, our results highlight the role of the C-terminus for A_2A_R and A_1_R in G-protein coupling, but not in G-protein specificity.

## Figures and Tables

**Table 1:** List of adenosine receptor high-resolution structures. Agonists are underlined.

Receptor	Year	Resolution (Å)	Expression Host	Ligand	Modification				Reference
					Chimera	Stabilization	Thermo-Stabilization	Truncation	
**A_2A_R**	2008	2.6	*S. frugiperda*	ZM241385	X			X	Jaakola et al. [9]
	2011	2.71	*S. frugiperda*	UK-432097	X			X	Xu et al. [10]
	2011	2.6–3	*Trichoplusia ni*	Adenosine; NECA			X	X	Lebon et al. [11]
	2011	3.3–3.6	*S. frugiperda*	Caffeine; ZM241385; XAC			X	X	Dore et al. [12]
	2012	2.7–3.1	*P. pastoris*	ZM241385		X		X	Hino et al. [13]
	2012	3.27–3.34	*S. frugiperda*	Novel compounds			X	X	Congreve et al. [14]
	2012	1.8	*S. frugiperda*	ZM241385	X			X	Liu et al. [15]
	2015	2.6	*Trichoplusia ni*	CGS21680			X	X	Lebon et al. [16]
	2016	1.72–2.2	*Trichoplusia ni*	ZM241385 and 4 novel compounds	X		X	X	Segala et al. [17]
	2016	3.4	*Trichoplusia ni*	NECA	X	X		X	Carpenter et al. [18]
	2016	1.9–2.5	*S. frugiperda*	ZM241385	X			X	Batyuk et al. [19]
	2017	3.5	*S. frugiperda*	Novel compound	X			X	Sun et al. [20]
	2017	3.2	*S. frugiperda*	ZM241385	X			X	Martin-Garcia et al. [21]
	2017	2.8	*S. frugiperda*	ZM241385	X			X	Melnikov et al. [22]
	2017	2–2.8	*Trichoplusia ni*	Theophylline; caffeine; PSB36	X		X	X	Cheng et al. [23]
	2017	1.7–2.14	*Trichoplusia ni*	ZM241385	X		X	X	Weinert et al. [24]
	2018	2.35	*S. frugiperda*	ZM241385	X		X	X	Broecker et al. [25]
	2018	2.51	*P. pastoris*	ZM241385	X			X	Eddy et al. [26]
	2018	1.87–3.1	*Trichoplusia ni*	Theophylline, ZM241385, Vipadenant, LUAA47070, Tozadenant and 2 novel compounds	X		X	X	Rucktooa et al. [27]
	2018	2.6–2.9	*P. pastoris*	UK-432097	X			X	White et al. [28]
	2018	4.11	*Trichoplusia ni*	NECA	X	X		X	Garcia-Nafria et al. [29]
	2019	4.2	*S. frugiperda*	ZM241385	X			X	Martin-Garcia et al. [30]
	2019	2.25	*S. frugiperda*	ZM241385	X			X	Shimazu et al. [31]
	2019	1.85	*S. frugiperda*	ZM241385	X			X	Ishchenko et al. [32]
	2020	1.92–2.13	*Trichoplusia ni*	Novel Ligands	X		X	X	Jespers et al. [33]
	2020	2	*S. frugiperda*	ZM241385	X			X	Lee et al. [34]
	2020	2	*Trichoplusia ni*	AZD4635	X		X	X	Borodovsky et al. [35]
	2020	1.8–2	*S. frugiperda*	ZM241385	X			X	Ihara et al. [36]
**A_1_R**	2017	3.2	*S. frugiperda*	DU172	X			X	Glukhova et al. [37]
	2017	3.3	*Trichoplusia ni*	PSB36	X		X	X	Cheng, et al. [23]
	2018	3.6	*Trichoplusia ni*	Adenosine and DU172	X				Draper-Joyce et al. [38]

**Table 2:** List of yeast strains used.

Yeast Strain	G Protein	Last 5 Amino Acids at C-Terminal	Equivalent Human Gα
MMY12, BY4741	Gpa1	KIGII^COOH^	GPA1 (yeast)
MMY14	Gpa1-Gαq(5)	EYNLV^COOH^	GNAQ, GNA11
MMY16	Gpa1-Gα16(5)	EINLL^COOH^	GNA15, GNA16
MMY19	Gpa1-Gα12(5)	DIMLQ^COOH^	GNA12
MMY20	Gpa1-Gα13(5)	QLMLQ^COOH^	GNA13
MMY21	Gpa1-Gα14(5)	EFNLV^COOH^	GNA14
MMY22	Gpa1-Gαo(5)	GCGLY^COOH^	GNAO
MMY23, CY13393	Gpa1-Gαi1(5)	DCGLF^COOH^	GNAI1, GNAI2, GNAT1, GNAT2, GNAT3
MMY24	Gpa1-Gαi3(5)	ECGLY^COOH^	GNAI3
MMY25	Gpa1-Gαz(5)	YIGLC^COOH^	GNAZ
MMY28, CY13399	Gpa1-Gαs(5)	QYELL^COOH^	GNAS, GNAL

**Table 3:** List of plasmids used for receptor expression in yeast and mammalian cells. Yeast expression plasmids contain an N-terminal leader sequence (PP) to improve receptor expression and trafficking to the plasma membrane [71].

Name	Plasmid
ARJ001	pRS316 pGal_1-10_ PP A_1_R mCit cyc_t_
ARJ002	pRS316 pGal_1-10_ PP A_1_/A_2A_R mCit cyc_t_
ARJ051	pRS316 pGal_1-10_ PP A_1_/A_2A_Δ316R mCit cyc_t_
ARJ030	pRS316 pGal_1-10_ PP A_2A_R mCit cyc_t_
ARJ057	pRS316 pGal_1-10_ PP A_2A_Δ316R mCit cyc_t_
ARJ194	pCEP4 A_1_R
ARJ195	pCEP4 A_1_/A_2A_R
ARJ196	pCEP4 A_1_/A_2A_Δ316R
CM001	pCEP4 A_2A_R
CM002	pCEP4 A_2A_Δ316R
ARJ073	pRS316 pGal_1-10_ PP pFus1 mCherry pTEF-kanR2-tTEF Fus1_t_ cyc_t_
ARJ172	pRS316 pGal_1-10_ PP pFus1 mCherry pTEF-hphMx6-tTEF Fus1_t_ cyc_t_
